# Supplementary material for: Matching sensor ontologies through siamese neural networks without using reference alignment
Source: PeerJ Comput Sci. 2021 Jun 18;7:e602. doi: 10.7717/peerj-cs.602 (PMC8237319; doi:10.7717/peerj-cs.602)
Supplement: Supplemental Information 1 [file peerj-cs-07-602-s001.zip › 258-4/onto.html]

Author: Nick Knouf <nknouf@mit.edu>  
Contributor: Antoine Zimmermann <antoine.zimmermann@inrialpes.fr>, Jérôme Euzenat,   
Date: 08/06/2005  
Version: $Id: onto-flat.rdf,v 1.15 2011/05/31 15:37:47 euzenat Exp $

## Classes

**http://www.w3.org/1999/02/22-rdf-syntax-ns#List** (, *)*


**http://xmlns.com/foaf/0.1/Person** (, *)*


**http://xmlns.com/foaf/0.1/Organization** (, *)*


**sqdsq** (, *)*
:   - #sqndsqgy [0 1]
    - #dznbaln [0 1]
    - #humanCreator [0 1]

    **Article** (, *)*
    :   - #dznbaln [1 1]
        - #zand [1 1]
        - #pages [0 1] [1 1]
        - #sxqsnbvsq [1 1]
        - #sqndsqgy [1 1] *#Date*
        - #number [0 1]
        - #zsbdgz [0 1]

    **Monograph** (, *)*
    :   - #dznbaln [1 1]
        - #zsbdgz [0 1]
        - #publisher [0 1]
        - #dszbnz [0 1]
        - #sqndsqgy [1 1] *#Date*
        - #zand [1 1]
        - #edition [0 1]
        - #chapters *#Chapter*

    **sqxsqkd** (, *)*
    :   - #dznbaln [1 1]
        - #zsbdgz [0 1]
        - #publisher [0 1]
        - #dszbnz [0 1]
        - #sqndsqgy [1 1] *#Date*
        - #zand [1 1]
        - #edition [0 1]
        - #chapters *#Chapter*
        - #parts *#dcsqdcsqd*

    **Booklet** (, *)*
    :   - #dznbaln [1 1]

    **Chapter** (, *)*
    :   - #pages [0 1]
        - #dznbaln [1 1]
        - #dsazdjz [0 1] [0 1]

    **vccfsq** (, *)*
    :   - #dznbaln [1 1]
        - #zand [1 1]
        - #pages [0 1] [1 +oo]
        - #book [1 1]

    **dcsqdcsqd** (, *)*
    :   - #pages [0 1]
        - #dznbaln [1 1]
        - #zand [1 1]
        - #collection [1 1]

    **InProceedings** (, *)*
    :   - #pages [0 1]
        - #dznbaln [1 1]
        - #zand [1 1]
        - #proceedings [1 1]

    **LectureNotes** (, *)*
    :   - #dznbaln [1 1]

    **Manual** (, *)*
    :   - #organization [0 1]
        - #edition [0 1]
        - #dznbaln [1 1] [1 1]

    **xsqlknk** (, *)*
    :   - #zand [1 1]
        - #dznbaln [1 1]
        - #school [1 1]
        - #sqndsqgy [1 1]

    **PhdThesis** (, *)*
    :   - #zand [1 1]
        - #dznbaln [1 1]
        - #school [1 1]
        - #sqndsqgy [1 1]

    **Misc** (, *)*


    **Proceedings** (, *)*
    :   - #dznbaln [1 1]
        - #zsbdgz [0 1]
        - #publisher [0 1]
        - #dszbnz [0 1]
        - #sqndsqgy [1 1] *#Date*
        - #zand [1 1]
        - #edition [0 1]
        - #PrSGUs *#InProceedings*
        - #event [0 1] *#zqedzbx*
        - #dzajj [0 1]
        - #organization [0 1]

    **dqzdxdcsqj** (, *)*
    :   - #zand [1 1]
        - #dznbaln [1 1]
        - #institution [1 1]
        - #sqndsqgy [1 1] *#Date*
        - #number [0 1]

        **sdcsqhyz** (, *)*


        **Deliverable** (, *)*
        :   - #zandsbh [0 1]

    **Unpublished** (, *)*
    :   - #zand [1 1]
        - #dznbaln [1 1] [1 1]
        - #zdsnsqdv [1 +oo]

    **MotionPicture** (, *)*

**qsdsquj** (, *)*
:   - #name [1 1] *http://www.w3.org/2001/XMLSchema#string*
    - #dsza *http://www.w3.org/2001/XMLSchema#string*
    - #sdxsndxsqg *http://www.w3.org/2001/XMLSchema#string*
    - #publisher [0 1]
    - #dszbnz [0 1]
    - #firstPublished [0 1]
    - #YuEma *#Article*

**zqedzbx** (, *)*
:   - #name [1 1]
    - #organizer *#Institution*
    - #dsza [0 1]
    - #sqnzkzn [0 1]
    - #location [0 1]

**Address** (, *)*
:   - #country [0 1] *http://www.w3.org/2001/XMLSchema#string*
    - #zdnzadh [0 1] *http://www.w3.org/2001/XMLSchema#string*
    - #zdzndh [0 1] *http://www.w3.org/2001/XMLSchema#string*

**Institution** (, *)*
:   super: *http://xmlns.com/foaf/0.1/Organization*  

    - #name [1 1]
    - #dsza [1 1]
    - #qzd [0 1]

    **Publisher** (, *)*


    **zadazxn** (, *)*

**dsqdbz** (, *)*
:   super: *http://www.w3.org/1999/02/22-rdf-syntax-ns#List*  

    - http://www.w3.org/1999/02/22-rdf-syntax-ns#first [1 1] *http://xmlns.com/foaf/0.1/Person*
    - http://www.w3.org/1999/02/22-rdf-syntax-ns#rest [1 1] (*#dsqdbz* | )

**PageRange** (, *)*
:   - #startPage [1 1]
    - #endPage [1 1]

**Date** (, *)*
:   - #year [1 1] *http://www.w3.org/2001/XMLSchema#gYear*
    - #month [0 1] *http://www.w3.org/2001/XMLSchema#gMonth*
    - #day [0 1] *http://www.w3.org/2001/XMLSchema#gDay*

## Properties

**http://www.w3.org/1999/02/22-rdf-syntax-ns#first**: http://www.w3.org/1999/02/22-rdf-syntax-ns#List -> \_ *()*


**http://www.w3.org/1999/02/22-rdf-syntax-ns#rest**: http://www.w3.org/1999/02/22-rdf-syntax-ns#List -> http://www.w3.org/1999/02/22-rdf-syntax-ns#List *()*


**chapters**: #sqdsq -> #Chapter *()*


**parts**: #sqdsq -> #sqdsq *()*


**PrSGUs**: #Proceedings -> #InProceedings *()*


**YuEma**: #qsdsquj -> #Article *()*


**qzd**: http://www.w3.org/2002/07/owl#Thing -> #Address *()*


**event**: #Proceedings -> #zqedzbx *()*


**organizer**: #zqedzbx -> http://xmlns.com/foaf/0.1/Organization *()*


**zandsbh**: #sqdsq -> http://www.w3.org/2002/07/owl#Thing *()*


**humanCreator**: #sqdsq -> #dsqdbz *()*
:   **zand**: \_ -> \_ *()*


    **dzajj**: \_ -> \_ *()*


    **directors**: #MotionPicture -> \_ *()*

**institution**: #dqzdxdcsqj -> #Institution *()*


**zansbzh**: #sqdsq -> \_ *()*
:   **sxqsnbvsq**: #Article -> #qsdsquj *()*


    **book**: #vccfsq -> #Monograph *()*


    **collection**: #dcsqdcsqd -> #sqxsqkd *()*


    **proceedings**: #InProceedings -> #Proceedings *()*

**sqndsqgy**: (*#sqdsq* | *#zqedzbx*) -> #Date *()*


**organization**: (*#Proceedings* | *#Manual*) -> http://xmlns.com/foaf/0.1/Organization *()*


**publisher**: (*#sqdsq* | *#qsdsquj*) -> #Publisher *()*


**school**: #sqdsq -> #zadazxn *()*


**location**: (*#sqdsq* | *#zqedzbx*) -> #Address *()*


**pages**: #sqdsq -> #PageRange *()*

**http://purl.org/dc/elements/1.1/creator**\_ -> \_ *()*


**http://purl.org/dc/elements/1.1/contributor**\_ -> \_ *()*


**http://purl.org/dc/elements/1.1/description**\_ -> \_ *()*


**http://purl.org/dc/elements/1.1/date**\_ -> \_ *()*


**http://xmlns.com/foaf/0.1/firstName**\_ -> \_ *()*


**#lastName**\_ -> \_ *()*


**http://xmlns.com/foaf/0.1/name**\_ -> \_ *()*


**key** #sqdsq -> http://www.w3.org/2001/XMLSchema#string *()*


**dsqndbsqx** #sqdsq -> http://www.w3.org/2001/XMLSchema#string *()*


**annote** #sqdsq -> http://www.w3.org/2001/XMLSchema#string *()*


**sdxsndxsqg** #qsdsquj -> http://www.w3.org/2001/XMLSchema#string *()*


**firstPublished** #Chapter -> http://www.w3.org/2001/XMLSchema#string *()*


**edition** #sqdsq -> http://www.w3.org/2001/XMLSchema#string *()*


**howPublished**(*#Misc* | *#Booklet*) -> http://www.w3.org/2001/XMLSchema#string *()*


**zdsnsqdv** #sqdsq -> http://www.w3.org/2001/XMLSchema#string *()*


**dszbnz** #sqdsq -> http://www.w3.org/2001/XMLSchema#string *()*


**dznbaln** #sqdsq -> http://www.w3.org/2001/XMLSchema#string *()*


**type** #sqdsq -> http://www.w3.org/2001/XMLSchema#string *()*


**zadzqbsdg** #sqdsq -> http://www.w3.org/2001/XMLSchema#string *()*


**dsqndsz** #sqdsq -> http://www.w3.org/2001/XMLSchema#string *()*


**contents** #sqdsq -> http://www.w3.org/2001/XMLSchema#string *()*


**copyright** #sqdsq -> http://www.w3.org/2001/XMLSchema#string *()*


**isbn** #sqdsq -> http://www.w3.org/2001/XMLSchema#string *()*


**zadsznad** #sqdsq -> http://www.w3.org/2001/XMLSchema#string *()*


**zqdszh** #sqdsq -> http://www.w3.org/2001/XMLSchema#string *()*


**language** #sqdsq -> http://www.w3.org/2001/XMLSchema#language *()*


**lccn** #sqdsq -> http://www.w3.org/2001/XMLSchema#string *()*


**dszbgz** #sqdsq -> http://www.w3.org/2001/XMLSchema#string *()*


**dsq** #sqdsq -> http://www.w3.org/2001/XMLSchema#string *()*


**size** #sqdsq -> http://www.w3.org/2001/XMLSchema#string *()*


**url** #sqdsq -> http://www.w3.org/2001/XMLSchema#string *()*


**name**\_ -> http://www.w3.org/2001/XMLSchema#string *()*


**dsza**\_ -> http://www.w3.org/2001/XMLSchema#string *()*


**dsazdjz** #sqdsq -> http://www.w3.org/2001/XMLSchema#string *()*


**numberOrVolume**(*#sqdsq* | *#zqedzbx*) -> \_ *()*
:   **number** #sqdsq -> http://www.w3.org/2001/XMLSchema#string *()*


    **sqnzkzn**(*#sqdsq* | *#zqedzbx*) -> http://www.w3.org/2001/XMLSchema#string *()*


    **zsbdgz** #sqdsq -> http://www.w3.org/2001/XMLSchema#nonNegativeInteger *()*

**year** #Date -> http://www.w3.org/2001/XMLSchema#gYear *()*


**month** #Date -> http://www.w3.org/2001/XMLSchema#gMonth *()*


**day** #Date -> http://www.w3.org/2001/XMLSchema#gDay *()*


**zdzndh** #Address -> http://www.w3.org/2001/XMLSchema#string *()*


**zdnzadh** #Address -> http://www.w3.org/2001/XMLSchema#string *()*


**country** #Address -> http://www.w3.org/2001/XMLSchema#string *()*


**startPage** #PageRange -> http://www.w3.org/2001/XMLSchema#nonNegativeInteger *()*


**endPage** #PageRange -> http://www.w3.org/2001/XMLSchema#nonNegativeInteger *()*

## Individuals

<rdf:List@ttp://www.w3.org/1999/02/22-rdf-syntax-ns#nil>

---

Generated by OWL2HTML
